# Recent Advances in Aqueous-Phase Catalytic Conversions of Biomass Platform Chemicals Over Heterogeneous Catalysts

**DOI:** 10.3389/fchem.2019.00948

**Published:** 2020-02-07

**Authors:** Xiaoxian Li, Lilong Zhang, Shanshan Wang, Yulong Wu

**Affiliations:** ^1^Institute of Nuclear and New Energy Technology, Tsinghua University, Beijing, China; ^2^Laboratory of Advanced Reactor Engineering and Safety of Ministry of Education, Institute of Nuclear and New Energy Technology, Tsinghua University, Beijing, China

**Keywords:** biomass, aqueous phase, heterogeneous catalysis, dehydration, hydrogenation, oxidation, isomerization, ketonization

## Abstract

A series of biomass-derived platform molecules, such as glucose, furans, levulinic acid, 5-hydroxymethylfurfural, and acetic acids, can be converted into a variety of value-added chemicals through catalytic transformations that include dehydration, hydrogenation, oxidation, isomerization, reforming, ketonization, and aldol condensation over heterogeneous catalysts. Aqueous-phase processing is an important issue and a great challenge for the heterogeneous catalytic conversion of biobased chemicals due to the high water content of the biomass and the formation of water during the transformation process. In this paper, heterogeneous catalysts that are applicable to the aqueous-phase conversion process of biomass platform chemicals, including noble metal catalysts, non-noble metal catalysts, bimetallic catalysts, metal oxides, and zeolite, are introduced, and a comprehensive evaluation of the catalyst performance, including the catalytic activity, stability, and regeneration performance of different kinds of heterogeneous catalysts, are made. Besides, we highlighted the effect of water on heterogeneous catalysts and the deactivation mechanism in the aqueous phase. Beyond this, several catalytic mechanisms of aqueous-phase conversion over heterogeneous catalysts are summarized in order to help understand the reaction process on the surface of catalysts in the aqueous phase, so as to design targeted catalysts. At last, a prospect of biobased chemicals and fuels is forecasted.

## Introduction

**Figure 1:**
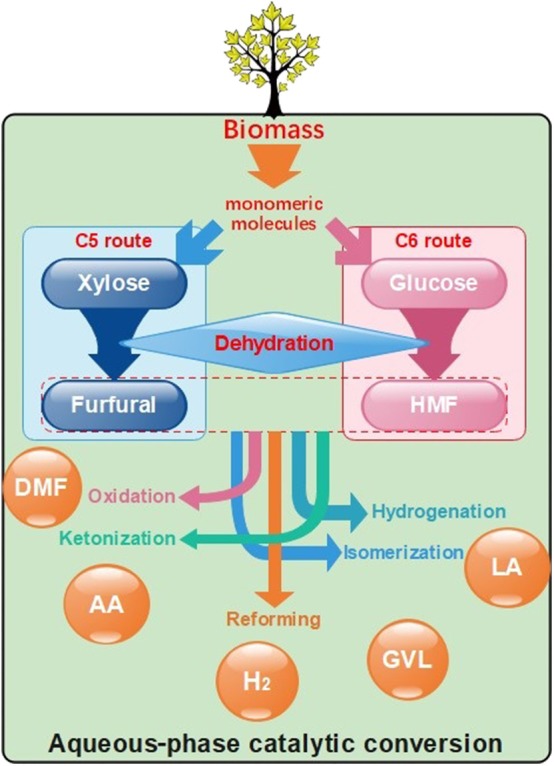
A general conversion route of biomass.

With the depletion of traditional fossil energy and its environmental adverse impact, alternative energy sources that have good renewability and environment friendliness are needed (Tang et al., 2017). Among all of the alternative resources, biomass is considered to be one of the most potential competitors not only as fuels but also as chemical intermediates (Dusselier et al., 2013) because of the rich abundant and good sustainability. There is no doubt that biofuels are promising products of the utilization of biomass, which have a huge market demand; however, the discussion on the conversion of biomass into biofuels is not within the scope of this work. Despite the fact that simple edible starting materials like starch and sugars can easily be converted into valuable products (Binder and Raines, 2009), this pathway is not perceived as a promising approach because it competes with food production, directly, or indirectly (Sheldon, 2014). In contrast, lignocellulose, which is composed of cellulose, hemicellulose, and lignin (Luterbacher et al., 2014), is edible, although relatively difficult to process. Similar to the petrifaction industry, the biomass-based industry can achieve economic feasibility by producing chemical platform molecules via several routes as shown in Figure 1, and then further converting them into valuable chemicals through biorefinery (Serrano-Ruiz et al., 2011). These platform compounds often contain a high degree of oxygenated groups, which can exert negative effects while converting them into fuels. On the other hand, the high oxygen content makes them water-soluble; therefore, it is possible to convert those chemicals through aqueous-phase processes at mild temperature and pressure. Besides, waters are produced either by biomass itself or by the process of reducing the oxygen content; this also provides a beneficial effect on the aqueous-phase conversion of biomass compounds.

During a general conversion route of lignocellulose, monomeric molecules such as xylose and glucose are firstly produced through the fermentation process (Menon and Rao, 2012). Subsequently, the key intermediate products, furfural, and 5-hydroxymethylfurfural (HMF), can be produced through dehydration of pentoses and hexoses, respectively (Chheda et al., 2007). These chemicals can be further processed into a variety of useful platform chemicals like levulinic acid (LA) or 2,5-dimethylfuran (DMF) (Gallezot, 2012) by hydrogenation or oxidation. These substances can be subsequently processed into desired products by isomerization (Roman-Leshkov et al., 2010), reforming (Cortright et al., 2002), ketonization (Pham et al., 2013), esterification (Fernandes et al., 2012), and aldol condensation (Patil and Lund, 2011). In the aqueous phase, the produced water has a lower effect on the conversion process. Heterogeneous catalysts, because of their excellent separation, are of good prospects for development. Obviously, the activity and stability of catalysts play a significant role in the process. For heterogeneous catalysts, properties like activity and selectivity depend on the surface area, pore structures, and acid sites, and stability and recyclability depend on water tolerance and hydrophobicity. Many reactions have been achieved in the gas phase or the organic solvent phase before; however, most catalysts cannot play full catalytic ability in the aqueous phase; the most common is deactivated rapidly, because of the degradation of catalysts caused by the corrosive effects of H^+^ or OH^−^ ions and dissolved organic carbon in water (Han Y. L. et al., 2016). Thus, it is necessary to improve the stability of heterogeneous catalysts in the aqueous phase by improving their surface hydrophobicity through doping of the support with heteroatoms, surface modification, thin-film coatings, or other ways (Ravenelle et al., 2011). In that case, increasing the hydrophobic character of catalysts through the increase in the Si/Al ratio of zeolites (Xiong et al., 2014), or using appropriate hydrophobic supports, such as metal oxide and carbon materials, can be used to improve the activity and hydrothermal stability (Ravenelle et al., 2011).

Several reviews have been published about reactions of biomass platform chemicals (Irshad et al., 2019; Liu et al., 2019; Luo W. H. et al., 2019). This field has broad prospects because of the demand for renewable energy resources. Furthermore, water can act not only as a solvent but also as a reactant, hydrogen source, or catalyst, which is quite different from the relatively inert organic solvents such as toluene and dioxane. Thus, catalysts show completely different activities and stabilities in the aqueous phase, organic phase, or gas phase, so that the aqueous-phase conversions are worth to be discussed in detail. The purpose of this review is to discuss the heterogeneous catalytic conversion in the aqueous phase of biomass platform chemicals, expect to provide a general understanding of this field, and give possible direction for developing novel catalysts or methods to the reactions. The following explanation starts from three aspects: species of biomass platform chemicals, advantages, and challenges of aqueous-phase conversion, and the characteristics of heterogeneous catalysts.

### Biomass Platform Chemicals

First of all, the types of biomass platform chemicals should be identified. These platform molecules were initially selected by the US Department of Energy (DOE) in 2004 (Werpy and Petersen, 2004) and revisited by Bozell and Petersen (2010). The original list includes carbon 1,4-diacids, 2,5-furan dicarboxylic acid (FDCA), 3-hydroxy propionic acid (3-HPA), aspartic acid, glucaric acid, glutamic acid, itaconic acid, LA, 3-hydroxybutyrolactone, glycerol, sorbitol, and xylitol/arabinitol. In the course of further research and development, some compounds, such as organic acids like aspartic acid, glucaric acid, glutamic acid, and itaconic acid, had been neglected and gradually faded out of sight (Bozell and Petersen, 2010); in contrast, some other compounds were carried out because of the demand arising from the progress of transformation technology. In recent years, research on biomass platform molecules includes the following: ethanol, furans (including furfural and HMF), glycerol, lactic acid, succinic acid, LA, sorbitol, and xylitol. Some of these molecules are produced by fermentation; some of them are converted from C_5_ or C_6_ sugars (Climent et al., 2014). The similar point of these chemicals is that they all have a large source and a variety of transformation methods.

### Advantages and Challenges of the Aqueous-Phase Conversion of Biomass Platform Chemicals

As previously mentioned, the aqueous-phase conversion is a feasible strategy for biomass utilization, because of the high dissolvability, easiness of separation, and green color and nontoxicity (Climent et al., 2014) of water as the solvent. Despite water being not the only option as a green solvent, it can be one of the most promising and competitive solvents because of its widespread presence in biomass sources. Moreover, water can also participate in the reaction through multiple ways, such as a hydrogen donor in the hydrogenation process (Hu et al., 2014) or as one of the reactors in ring opening and isomerization reaction (Choudhary et al., 2013). The most noteworthy is the effect of hydrogen bonds; water as a solvent can significantly accelerate the transformation of H atoms from the bulk phase to the catalyst surface through a hydrogen bond network composed of water, which is called the Grotthuss mechanism (Agmon, 1995). However, the hydrothermal stability of heterogeneous catalysts has been one of the critical factors restricting the development of biomass aqueous-phase transformation technology (Huo et al., 2018). Although this stability can be improved through particular treatments, such as modification with metals, coating, or silanization (Gayubo et al., 2010), these treatments are often observed to affect other properties of catalysts. Therefore, it is necessary to expand the development of the transformation of biomass resources and the efficient utilization technology in the aqueous phase.

### Heterogeneous Catalysts in the Aqueous Phase

According to the different physical and chemical properties of the reactants, different kinds of catalysts are required. In general, these catalysts can be divided into homogeneous and heterogeneous catalysts. Homogeneous catalysts have the advantage of high solubility, which makes them better to contact with the reactants, to show higher activity or milder reaction conditions (Shylesh et al., 2010). However, homogeneous catalysts faced difficulty in catalyst recovery and separation with the products, which increases their cost, and that becomes one of the bottlenecks of the industry. On the other hand, with the increasing environmental and economic demands for sustainability (Shylesh et al., 2010), heterogeneous catalysts, with the advantages of environment friendliness, easy separation, and great reusability, are more in line with the needs of technological development in recent days. The heterogeneous catalysts that are applied for the aqueous-phase conversion of biomass platform chemicals are usually porous materials with high surface activity and specific surface areas, such as alumina, silica, carbon materials, and metal oxides (Wu K. J. et al., 2016). Because of the foundation of the petrochemical industry, traditional heterogeneous catalysts are mainly used in the conversion of hydrocarbons in the gas phase, which enables them to be stable at high temperatures and to adapt to non-polar compounds (Rinaldi and Schuth, 2009). As highlighted above, however, the aqueous-phase conversions are quite different, and that places new requirements for heterogeneous catalysts.

To date, biomass platform chemicals' heterogeneous catalytic reactions in the aqueous phase, such as dehydration, hydrogenation, oxidation, isomerization, reforming, ketonization, and aldol condensation, have been studied. In the following sections, we will highlight the recent advances of heterogeneous catalysts for these reactions in the aqueous phase.

### Production of Biomass Platform Chemicals From Biomass

As described above, biomass platform chemicals could be various in different types. A common way of classification is through the carbon number, which is closely related to its biomass source. Therefore, compared with the conversion and utilization of biomass, the production process of biomass is also worthy of attention. Because of the limitation of the focus and length of this paper, too much discussion may not be added here, but the most basic discussion is necessary.

Biomass platform chemicals are originally produced by wood or algae through thermochemical conversion processes. Wood generally contains cellulose (40–50%), hemicellulose (10–30%), and methyl cellulose (20–30%). According to different components, different biomass platform chemicals can be produced. Cellulose, which is formed by the D-glucose units, can be converted to glucose by the hydrolysis of β-1,4 glucan (Suganuma et al., 2008), and then goes through the C_6_ pathway to other platform chemicals such as sorbitol (Rey-Raap et al., 2019), 5-HMF (Yu and Tsang, 2017), or LA (Kang et al., 2018). In contrast, hemicellulose is not a single carbohydrate polymer. The hardwood hemicelluloses contain mostly xylans (Saha, 2003), which is the major source of xylose and C_5_ platform chemicals such as furfural (Luo Y. P. et al., 2019). Furans are a promising and important platform chemical because they are precursors of many other platforms as mentioned above. Therefore, the platform chemical system constructed by cellulose and hemicellulose has been widely studied.

The case of algae is quite different. The main components of algae are fat, polysaccharide, and protein (Khoo et al., 2019), which makes the problem more complicated. Though the current conventional way of utilization is to make it into bio-oil, the complex composition of algae also provides a possible way to separate each component and produce value-added chemicals (Harun et al., 2010); the most common are acetone, glycerol (Karimi et al., 2015), bioethanol (Dave et al., 2019), or other alcohols. In general, algae are not the main battlefield for the production of biomass platform chemicals.

## Dehydration

High-valued products of furan compounds such as furfural and HMF, generated from dehydration of renewable biomass platform chemicals of six-carbon ketose and pentose, have been identified as a primary and versatile renewable biomass feedstock. Dehydration reactions play vital roles in aqueous-phase catalytic processing to produce fine chemicals and liquid fuels from biomass-derived oxygenated hydrocarbons (Huber et al., 2004).

Xylose, glucose, and sorbitol as the most abundant renewable biomass platform chemicals can be transformed to several molecules such as HMF, furfural, levulinic ester, furfuryl alcohol, and isosorbide. Because of the rehydration and polymerization reactions, many by-products, such as organic acids and humins, were synthesized in glucose dehydration to HMF in the water phase (Delidovich et al., 2014).

In this part, we summarize recent advances in the heterogeneous reaction system for the catalytic production of furan compounds from dehydration of biomass platform chemicals (Wang et al., 2017). Solid acid catalysts are advantageous due to their economic promise, environmental viability, and recyclability for aqueous-phase dehydration. Thus, solid acid catalysts such as phosphate, nanosized mixed oxides, and zeolites have been investigated (Kobayashi et al., 2015).

### Phosphates

A great deal of effort was focused on the development of efficient, inexpensive, simple structure and easily separated heterogeneous solid acid phosphate catalysts, in order to solve the shortcomings of restricted large-scale use and complex preparation process. What's more, a phosphate catalyst containing both Lewis and Brønsted acidic sites and the synergistic effect of Lewis and Brønsted acid sites in the phosphate catalyst were demonstrated to be an efficient conversion of xylose to furfural. Typically, the catalyzed dehydration of xylose to furfural involved the isomerization of xylose to xylulose or lyxose, followed by dehydration to produce furfural. Lewis acid plays a critical role in the xylose-to-xylulose isomerization, while Brønsted acid is active for xylulose dehydration. On the other hand, for the dehydration of xylose to HMF or furfural, lots of studies have also confirmed that the conversion rate of xylose and the yield of HMF or furfural were greatly improved in the water/organic biphasic system by using the solid phosphate catalysts. For xylose dehydration to furfural, it is desirable to have a high ratio of Brønsted to Lewis acid sites to design efficient aqueous-phase dehydration catalysts. Lewis acid sites decrease furfural selectivity by catalyzing a side reaction between xylose and furfural to form humins. Fang et al. (2017) demonstrated that a high furfural yield of 54.9 mol% and a complete conversion (100%) of xylose were obtained at 180°C for 3 h in the aqueous phase by a bifunctional NbOPO_3_ catalyst. In the work reported by Xu et al. (2018), a furfural yield could reach 18% from the xylose at 160°C for 60 min under a CrPO_4_ catalyst in the aqueous phase. However, an excellent furfural yield is up to 88% at the same condition by CrPO_4_ catalysis in the water/tetrahydrofuran biphasic system. Moreover, after four catalytic cycles, a desired 47% furfural yield was still obtained.

### Nanosized Metal Oxides

In recent years, production of nanosized metal oxide catalysis material led to new innovations and improvements in science and industry. It has been confirmed that metal oxide nanoparticles with a large surface area such as CuO, ZnO, Ga_2_O_3_, and MgO exhibit good activity and selectivity for the dehydration of xylose to furfural (Kumar et al., 2015).

**Figure 2:**
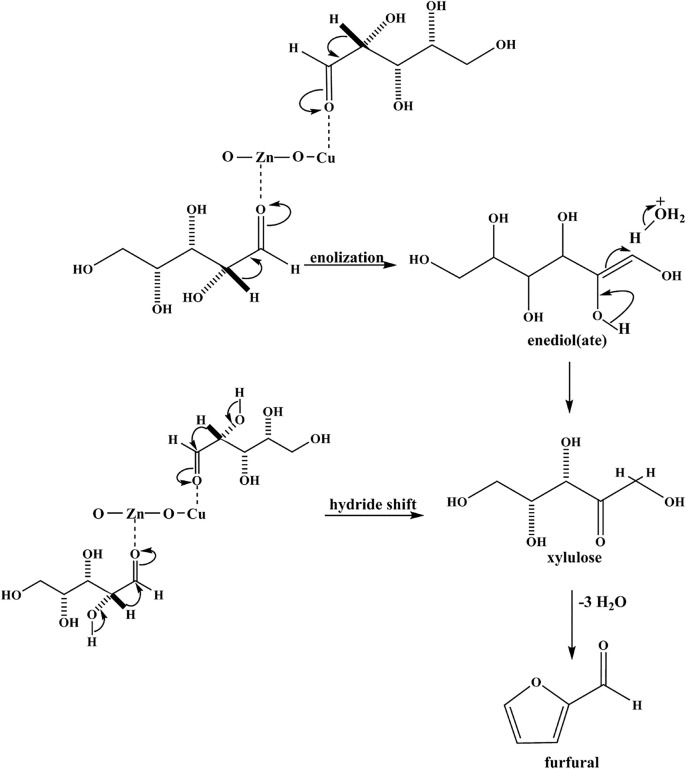
Possible reaction mechanism for the dehydration of xylose to furfural on Zn doped CuO catalyst. Reprinted from Mishra et al. (2019), with permission from Elsevier.

Zn doped CuO nanoparticles (Cu_0.89_Zn_0.11_O) synthesized by Gedanken group (Mishra et al., 2019) exhibit good activity and selectivity for the conversion of xylose to furfural, which enhances its catalytic activity and enables it to completely convert xylose and the 86% yield of furfural at 150°C within 12 h. The incorporation of Zn into CuO lattice resulted in a highly defective structure of the Zn doped CuO NPs, which provide more active sites for xylose to dehydrate. The Zn doped CuO NPs catalyst on the mechanism of dehydration of xylose to furfural is provided in Figure 2. In this mechanism, the reaction is in the forward direction via xylose isomerization with a 1,2-hydride shift. Then, xylulose is converted into an oxicarbenium ion by Zn and Cu, which act as Lewis acids. The subsequent deprotonation of these species produces an enol and further yields furfural by losing three molecules of water.

The catalytic activity of β-Ga_2_O_3_ nanorods was examined during the dehydration reaction of xylose to furfural, and results showed that β-Ga_2_O_3_ exhibits good activity and selectivity. The 94% yield of furfural is obtained at 150°C within 12 h (Kumar et al., 2016). The inherent characteristics of the stimuli and thermoresponsiveness for gallium-based oxide nanoparticles catalyst might be key factors leading to higher production of furfural from xylose.

Sn-based catalysts have demonstrated high activity for dehydration of glucose to platform chemicals, such as HMF and lactic acid. The catalytic results obtained by Sn supported on γ-Al_2_O_3_ (Sn/γ-Al_2_O_3_) catalyst concluded that the Lewis acidity offered by Sn oxides promotes the retro-aldol reaction pathway toward lactic acid, while the Lewis acidity offered by γ-Al_2_O_3_ enhances the synthesis of both HMF and lactic acid (Marianou et al., 2018). HMF yields of 12 and 88% glucose conversion were obtained in the aqueous phase at 150°C, 60 min, by Sn/γ-Al_2_O_3_. The highest HMF molar yield of 27.5% (at complete glucose conversion) was achieved. Lewis acid was used as a catalyst for the dehydration of glucose to produce versatile high value-added biochemicals and liquid biofuels.

### Zeolites

Zeolites with well-defined pores between 5 and 13Å are usually highly structured crystalline inorganic aluminosilicates, which show excellent thermal and chemical stability and mostly present Brønsted acidity giving them the ability to transform substrates into products, for instance, sorbitol dehydration into isosorbide, and glucose dehydration to prepare HMF (Kruger et al., 2012).

Sorbitol is one of the top 10 platform chemicals in biorefinery proposed by US DOE (Bozell and Petersen, 2010), and the most promising derivative of sorbitol is isosorbide. Isosorbide is used for treating glaucoma, brain hypertension, and Ménière's disease (Yemi and Mazza, 2019). The most common zeolites used in the dehydration of sorbitol into isosorbide are H-beta zeolites. Kobayashi et al. (2015) revealed that conversion of sorbitol to isosorbide by H-beta zeolites with a drastically high Si/Al ratio of 75 gives isosorbide in up to 76% yield at 127°C for 2 h.

In the comparison study of the catalytic performance for dehydration of D-xylose into furfural obtained with H-zeolites, Kim et al. (2011) carried out some contrast experiments by H-Y, H-mordenite, H-Ferrierite, H-ZSM, and H-beta. The highest furfural yield of 21.9% was obtained with H-Y in the presence of the aqueous solvent. They concluded that the D-xylose conversion and furfural yield generally decreased with an increasing Si/Al ratio of the H-zeolites.

Researchers have demonstrated that the zeolites are active in the glucose dehydration to prepare HMF. Mercedes revealed a glucose conversion of 57% and an HMF yield of 1.6% in the aqueous phase at 195°C, 30 min, using H-ZSM-5 zeolites with a Lewis/Brønsted molar ratio of 0.25. However, a conversion of 80% and an HMF yield of 42% were achieved by using a biphasic NaCl (20 wt%) aqueous solution/methyl isobutyl ketone system (Moreno-Recio et al., 2016). They corroborated that the catalytic activity is enhanced with the H-ZSM-5 zeolite introducing inorganic salt NaCl solution in the biphasic reaction medium.

The dehydration of xylose to furfural in aqueous solutions by a ZSM-5 zeolite catalyst was studied by O'Neill et al. (2009). They demonstrated that 46% furfural yield was produced at 200°C in an aqueous medium by H-ZSM-5 with a pore size of 1.2 nm. Therefore, a zeolite with a pore size around 0.8 nm, which ideally is close to xylose and furfural molecular sizes, would be more effective for the dehydration of xylose to furfural, as it would allow enough room for xylose to thread in and dehydrate to furfural.

Compared with microporous zeolites, mesoporous zeolites can provide excellent support because they are free from kinetic diffusion limitations in the case of larger molecules. Mesoporous zeolites, due to their good structural feature, high surface area, and relatively large pore size, have been widely studied for the dehydration of biomass platform chemicals, such as glucose dehydration to HMF. The most widely described reaction route of glucose to HMF consists of two steps: (1) isomerization of glucose to fructose in which Lewis acid sites play a significant role, and (2) dehydration of fructose to HMF; appropriate Brønsted acid sites are beneficial to the production of higher HMF yield. Jiang et al. designed and synthesized a zirconium doped mesoporous KIT-6 catalyst (Zr-KIT-6) for the dehydration of glucose into HMF. The maximum glucose conversion of 54.8% and HMF yield of 19.5% are obtained at 170°C after 3 h in an aqueous phase. In the biphasic MIBK–water system, 79.0% conversion of glucose and 34.5% HMF yield were optimally obtained (Jiang et al., 2018). This excellent result is mainly attributed to the highly distributed ZrO_2_ nanoparticles and multicoordinated Zr^4+^ species in Zr-KIT-6 samples.

**Figure 3:**
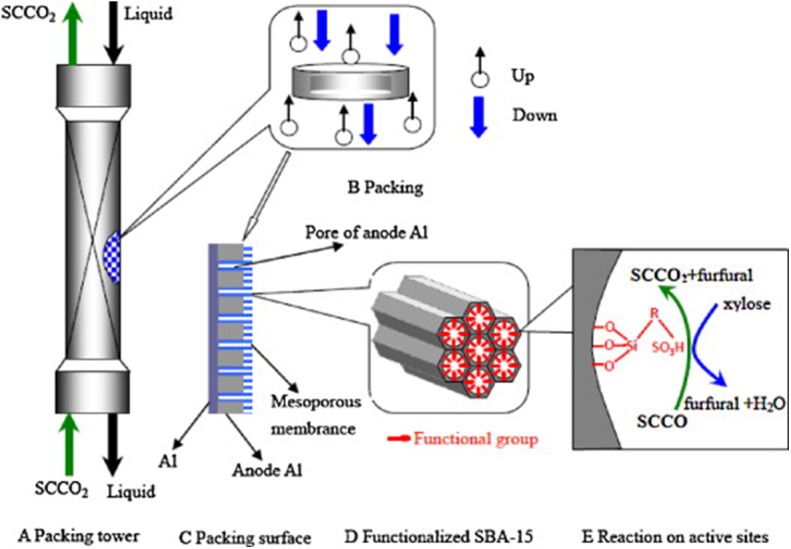
The scheme of solid acid catalyst packing and dehydration of xylose to furfural. Reprinted from Hua et al. (2013), with permission from Elsevier.

Mesoporous SBA-15 materials, because of their unique surface, pore structure with tunable uniform hexagonal channels, and hydrothermal stabilities, are a good candidate as a support for the dispersion of active centers. Wu's group (Shi et al., 2011a; Hua et al., 2013) designed synthesis SBA-15-SO_3_H and AAO/SBA-15-SO_3_H catalysts containing active centers of sulfoacid, a support of SBA-15 and substrate of porous alumina membrane (AAO). The conversion of xylose and selectivity of furfural were 90 and 70%, respectively, at 160°C for 4 h in a water/toluene two-phase system on both catalysts. A high reaction rate, mass transfer, and heterogeneous distribution of the active site of the AAO/SBA-15-SO_3_H catalyst are shown in Figure 3 (Hua et al., 2013).

Besides, Wu prepared a metal oxide loaded on the support of SBA-15 as Al-promoted ${\text{SO}}_{4}^{2-}$/ZrO_2_/SBA-15 catalysts (Shi et al., 2011b). The introduction of the Al stabilizes the tetragonal phase of the ZrO_2_ and thus increases the number and intensity of acid sites. The catalytic activity for dehydration of xylose to furfural was investigated. With increasing Al loading to 12%, the 98.7% xylose conversion and 53.4% furfural selectivity can be obtained at 160°C for 4 h in a H_2_O/toluene biphasic system.

In addition to the above catalysts, the relatively cheap and environmentally friendly ion-exchange resin was reported for the conversion of xylose to furfural. The result for the reaction of the xylose conversion rate is 71.8% and the furfural yield is 28.8% in water at 150°C for 16 h with Amberlyst 70 (specific surface area 36 m^2^ g^−1^ concentration of acid sites 2.65 mmol-H^+^ g^−1^, average pore diameter 22 nm) as the catalyst (Sato et al., 2019).

### Summary

Referring to the recently published literatures on the dehydration of biomass platform chemicals in the aqueous phase, the heterogeneous catalysts have been widely investigated. In this part, we have summarized the heterogeneous catalysts that have been relatively well studied in recent years including phosphate, nanosized metal oxides, and zeolites, which are mostly used for the dehydration of glucose, xylose, and sorbitol to furfural and isosorbide. For phosphate catalyst and zeolites, they have the common characteristic that the synergistic effect of Lewis and Brønsted acid sites with a high ratio of Brønsted to Lewis acid sites was demonstrated to be efficient and has superior activity to transform xylose and glucose into furfural. Nanosized metal oxides exhibit good activity and selectivity for the dehydration of xylose to furfural. From the current results, the highest conversion rate can reach ~100% by the phosphate of NbOPO_3_ and the mesoporous SBA-15 catalyst on the dehydration of xylose. On the other hand, the highest yield of furfural (94%) dehydration from xylose is obtained by the metal oxide of β-Ga_2_O_3_ nanorod. To sum up, the design and synthesis of a catalyst, which not only can convert reactants to 100% and achieve 100% yield but also has good hydrothermal stability, will be a difficult problem that we urgently need to solve.

## Hydrogenation

Most of the biomass platform chemicals, such as furfural or HMF, contain unsaturated bonds. Hydrogenation is needed in the process of the conversion to fine chemicals or bio-oil. The essence of hydrogenation, hydrogenolysis, and hydrodeoxygenation is the hydrogenation of C=O, C=C, or C–O bonds; generally, C=O bonds are more easily activated. In recent research of hydrogenation, VIII group metal catalysts, especially Ni, Pt, Pd, or Ru, are mostly studied; noble metal catalysts usually show higher catalytic activity; however, more works are demanded to stop the conversion at target products, instead of excessive hydrogenation. In this section, recent advances in heterogeneous catalysts for aqueous-phase hydrogenation of biomass platform chemicals are discussed in order to provide ideas for the design of new catalysts.

### Noble Metal Catalysts

Because of outstanding activity, selectivity, and stability, noble metal catalysts are considered to be one of the best hydrogenation catalysts. The high activity can be attributed to the special electron and band structure. The adsorption capacity of the substrate depends on the percentage of d orbital (d%) in the *spd* hybrid bonding orbit. In noble metals such as Ru, Pd, and Pt, the d% can be over 40% or even 50%.

Noble metal supported catalysts received the most extensive research because of the excellent hydrogenation activity in the aqueous phase. Under the existence of noble metal catalysts, the hydrogenation of furfural (Taylor et al., 2016) and LA (Dhanalaxmi et al., 2017) can reach nearly 100% conversion and selectivity. Li et al. (2016) compared the reaction mechanisms between Cu and group VIII metals of the conversion of furfural to furfural alcohol, showing that the different binding mode and lower activation barrier interpreted the higher activity of group VIII metal. Zhao et al. (2019) explained the effect of water of the same conversion through free energy calculations, illustrating that water helps surface charge separation to form solvated protons in water and electrons left in the metal reservoir, both of which reach the adsorbed reactants simultaneously (Zhao et al., 2019). The activation barrier of hydrogenation can be significantly reduced by the transformation of the proton through a hydrogen-bonded water network. Thus, it can be seen that the advantages of using the aqueous phase on hydrogenation are mainly manifested in hydrogen bonds. On the one hand, the binding energy of substrates can be decreased through hydrogen bonds with water. On the other hand, the proton transfers through hydrogen bonds between water molecules can accelerate the protonation of the carbonyl group.

The effect of support should not be neglected. Li S. P. et al. (2019) decorated Ru on the ultrathin anatase TiO_2_ nanosheets with exposed (001) facets, which proved that the different electronic and surface properties of support can strongly affect the activity of the catalysts. Ru/TiO_2_-n, which contains most (001) planes, showed the highest activity because of the presence of more Ru (0) on the surface. Abdelrahman et al. (2014) studied the mechanism of this reaction. LA firstly hydrogenated in the Ru sites, and then acid-catalyzed dehydration occurs homogeneously catalyzed by acid. Therefore, it is necessary to regulate acidity and alkalinity according to the reaction.

A characteristic of aqueous-phase hydrogenation is that it is a gas–liquid reaction that occurs at the liquid–solid interface, so the mass transfer restriction can be one of the major influencing factors. Under that influence, overcoming the mass transfer limitation can speed up the reaction process. Bagnato et al. (2018) synthesized a series of catalytic membranes to increase the phase interface for the selective hydrogenation of furfural to furfural alcohol. The use of Ru-based catalytic membrane reactor significantly reduced the H_2_ requirement and increased the turnover frequency. The furfural alcohol selectivity reached >99% under 70°C and 7 bar. However, it was observed that some of Ru clusters were lost after the reaction; it is necessary to find some more resistant metal support.

As reported by Yakabi et al. (2018), Pd/Al_2_O_3_ catalysts were synthesized for the hydrogenation of succinic acid to γ-butyrolactone (GBL). Among catalysts with different loads, 2 wt% Pd/Al_2_O_3_ prepared by co-precipitation achieved optimal performance. Transmission electron microscope (TME) images and particle size distributions show that this catalyst possessed a smaller mean particle size and a narrower particle size distribution. The particle size and dispersion of metal clusters can affect the catalytic activity, which are also reported by Upare et al. (2011); the Ru/C catalyst showed the best catalytic activity attributed to the higher dispersion.

In summary, not only particle size and dispersion of the metal active sites but also structure or even orientation of the support can affect the activity, selectivity, and stability of catalysts. The different impact of water on noble metals is also noticeable. Besides, the high price of noble metals is still a problem; using bimetallic catalysts or designing a new reactor with higher availability might be a way out.

### Non-noble Metal Catalysts

Because of the high price of noble metal catalysts, some of the non-noble metals that have similar chemical properties and catalytic activity, such as Fe, Co, Ni, or Cu, also received great attention.

Dutta et al. (2019) summarized Ni, Cu, and Zr supported catalysts for the conversion of LA to gamma-valerolactone (GVL) reported in the last 5 years. Some of them, such as Ni/Al_2_O_3_, Cu/ZrO_2_, or Cu/Al_2_O_3_, showed 100% conversion and >99% selectivity at harsh conditions (about 200°C, >30 bar H_2_ pressure). However, further research on catalyst stability and recyclability is still needed. The leach of metal can be attributed to the weak interplay between metals and supports, but stronger interplay may sacrifice dispersion and reduce the catalytic activity.

Cerium also showed fair activity in the hydrogenation reaction. Feng et al. (2019) reported a (CePO_4_)_0.16_/Co_2_P catalyst, which achieved 98.2% LA conversion and 97.1% GVL yield at 90°C, 4.0 MPa H_2_ in 1.5 h. Moreover, the catalyst continued to have high performance in at least five cycles, which proved that it can maintain long-term activity and stability under strongly acidic conditions.

In contrast, non-noble catalysts are used in the hydrogenation of furfural more frequently. Gong et al. (2018) reported two N-doped carbon nanotube-encapsulated metal nanoparticles called Ni@NCNTs-600-800 and Co@NCNTs-600-800, which showed excellent catalytic performance in the hydrogenation of furfural. The yield of furfural alcohol reached as high as 100% at 80°C and still active after six cycles. The high yield of deep hydrogenation products, tetrahydrofurfuryl alcohol or cyclopentanone, indicated that the catalyst has greater catalytic potential at higher temperature and pressure.

In summary, non-noble metal catalysts can reduce the cost of catalysts but faces the predicament of metal leaching for a long time. With the research in-depth, this problem is gradually being solved to a certain extent. Nevertheless, the superiority of noble metal catalysts is still reflected in terms of conversion, selectivity, or reaction conditions, such as temperature and pressure. In order to give full play to the advantages of high activity of noble metal catalysts and low cost of non-noble metal catalysts, a possible way to achieve that is to use bimetallic catalysts.

### Bimetallic Catalysts

In the directional selection of products, catalysts with appropriate activity are required frequently. In recent years, some bimetallic catalysts are reported. Some of them are non-noble metal catalysts modified with noble metals, and some of them are alloy metal catalysts. These kinds of catalysts are designed based on the structural and catalytic characteristics of different metals, thus improving the catalytic efficiency through synergism.

Qiu et al. (2019) reported a Ru prompted MoO_3−x_/C catalyst in the conversion of sorbitol into C_5_-C_6_ alkane, which showed the excellent activity of 87.3% yield. The production of alkanes depends on the relative rates on C–C and C–O bond cleavage (Li and Huber, 2010), so the concentration of metal to acid sites is the key to control the product distribution. The addition of Ru promoted the reduction of Mo to strengthen the hydrogenation capacity of it.

**Figure 4:**
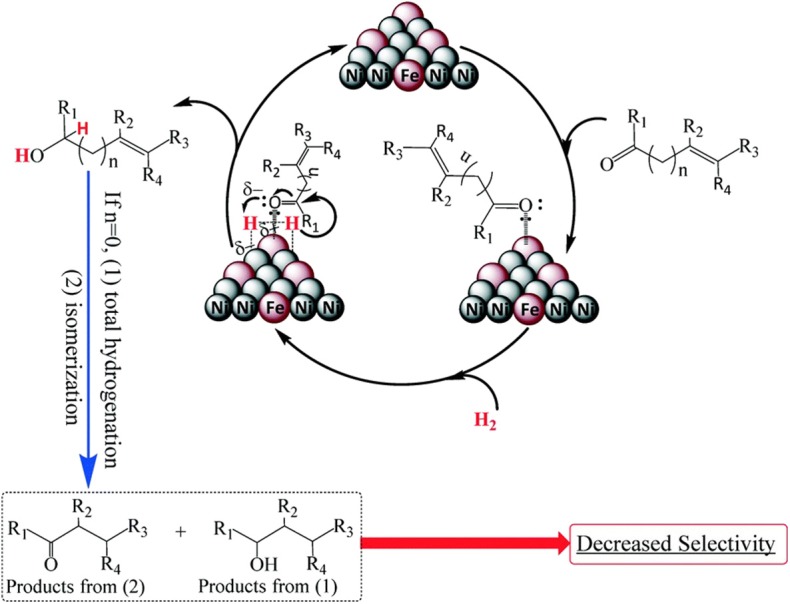
Plausible mechanism for the selective hydrogenation of unsaturated alcohols over Ni–Fe-based alloy. Used with permission of Royal Society of Chemistry, from Putro et al. (2017); permission conveyed through Copyright Clearance Center, Inc.

Putro et al. (2017) synthesized a Ni–Fe alloy catalyst by using a hydrothermal method that achieved high selectivity in the hydrogenation of furfural. The result suggested that the electron-deficient Fe can weaken and activate the C=O bonds through a side bonding interaction of the lone pair of oxygen in the carbonyl group, making π-complexing occur between the C=O bonds and the Ni atoms, as shown in Figure 4.

Using non-noble metal catalysts can reduce the cost, and the promotion of noble metal or other effective metals can optimize the performance of the catalyst. Hence, it can be a feasible technique of aqueous-phase conversion of biomass platform chemicals.

### Summary

Hydrogenation, which can reduce the unsaturation and oxygen content, is a very important conversion of biomass platform chemicals. The advantages of aqueous-phase hydrogenation are manifested in hydrogen bond, which can decrease the binding energy of substrates and accelerate the process of carbonyl group protonation. As far as the catalytic effects are concerned, noble metal catalysts present the best activity, stability, and recyclability, but they will cost more. Because many noble metal catalysts can achieve nearly 100% conversion, the higher activity cannot be reflected. Hence, more temperate reaction conditions and better stability are the current research direction. On the other hand, this also provides an opportunity for non-noble metal catalysts. Because of the congenital advantage in price, non-noble metal catalysts gained a lot of attention, especially in the study of the catalytic stability and recyclability to prevent the loss of metal, which is the dominant issue at present.

With the in-depth studies of the active sites of metal catalysts, the effects of the support structure and the interaction with metals are attracting more attention. The surface morphology and defects of support can strongly affect the dispersion and the stability of the combination of metal active sites. Moreover, the acid sites on the surface of support also play an important role in the conversion process. Different calcination temperature and acid or base treatment can affect the surface topography of support, but the effect on the activity and stability of catalysts is complicated. In general, treatment of acid or base brings the increase in active sites, which can enhance the catalytic activity but reduce the stability at the same time.

## Oxidation

In the traditional petrochemical industry, a wide range of commodity chemicals are produced via selective oxidation of petroleum-derived feedstock (Zope et al., 2010; Lanzafame et al., 2014; Kwon et al., 2016). Lignocellulose typically contains 40–45% (weight in dry base) oxygen content, which outclassed that of petroleum (He et al., 2014). These value-added molecules could potentially be more economical to produce platform chemicals containing oxygen elements from biomass rather than petroleum. So, catalytic oxidation of biomass into platform chemicals widely attracts scholars' attention.

**Figure 5:**
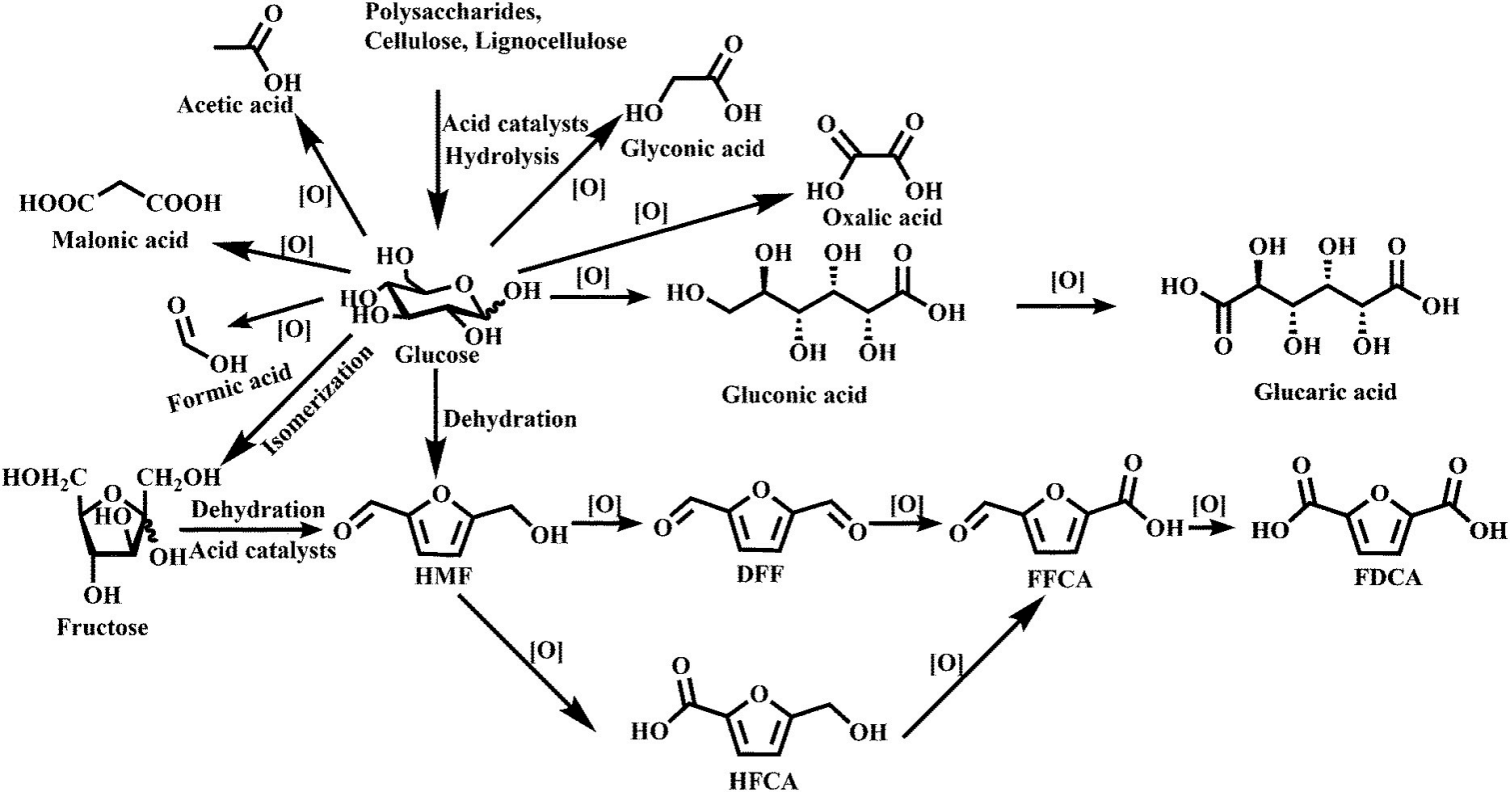
Chemicals produced by oxidation of glucose and glucose-derived molecules. Used with permission of Royal Society of Chemistry, from Zhang and Huber (2018); permission conveyed through Copyright Clearance Center, Inc.

Recently, many reports have been published about oxidation of biomass-derived compounds into value-added platform chemicals (Albonetti et al., 2015; Elliott et al., 2015; Huang et al., 2018; Zhang and Huber, 2018). Figure 5 shows that a wide variety of commodity platform chemicals can be produced from the catalytic oxidation of cellulose or carbohydrate-derived compounds.

### Direct Catalytic Conversion of Cellulose Into HFM

The necessary path of conversion from biomass into platform chemicals is cellulose hydrolysis and monosaccharide oxidation (Deng et al., 2014). It basically requires two steps: the acid-catalyzed hydrolysis of carbohydrates (cellulose or lignocellulose) into monosaccharides (glucose or xylose) and the oxidative cleavage of C–C bonds in monosaccharides into organic acid (Wang et al., 2018).

For the first step, liquid acid like sulfuric acid and hydrochloric acid was widely applied in the hydrolysis conversion of cellulose due to its high catalytic activity, acid strength, hydrogen ion releasing efficiency, and lower cost (Liu et al., 2013; Su et al., 2018). However, compared with liquid acid, solid acid has many advantages: (1) easy in separation and purification of the products; (2) stable catalysis effect under high temperature; and (3) aerobic oxidation can be controlled by the modified surface functional group of solid acid (Zhang and Zhao, 2009; Rinaldi et al., 2010). Yang and Pan (2016) found that a functional mesoporous polymeric catalyst bearing boronic acid as cellulose-binding groups and sulfonic acid as cellulose-hydrolytic groups was prepared, resulting in an excellent hydrolysis performance.

For monosaccharide oxidation, different oxidation degree has an essential influence on oxidation productions. Over-oxidation of glucose and xylose will lead to the complete combustion of monosaccharide to water and CO_2_, which are the thermodynamically favored products (Gliozzi et al., 2014). Thus, it is of critical importance that the selected catalyst systems control the degree of the reaction oxidation.

Hence, suitably functional catalysts combining both metal and acidic sites are necessary to combine the hydrolysis with oxidation steps into a one-pot reaction that converted polysaccharides into platform chemicals. The oxidative cleavage of C–C bonds is usually activated by metal catalysts. Polyoxometalates (POMs) with a formula of H_3n_PV_n_Mo_12n_O_40_ (HPA) contains Vanadium(V), which has acid sites that are considered as one effective catalysis for organic acid production from biomass with O_2_ (Wolfel et al., 2011). Zhang investigated a temperature-responsive heteropolyacid (H_2_PW_12_O_40_) catalyst. It was prepared by H_3_PW_12_O_40_ and choline chloride (ChCl) as catalysis-based material. And 75.0% HMF yield and 87.0% cellulose conversion were achieved under the condition of 140°C for 8 h in biphasic solvent system H_2_O and methyl isobutyl ketone (MIBK) (H_2_O/MIBK=1) in the one-pot transformation of cellulose to HMF (Teong et al., 2014). By contrast, with the homogeneous H_3_PW_12_O_40_, the excellent HMF yield may result from the higher Brønsted acidity (Choudhary et al., 2012). Li reported an Nb/carbon catalyst (Nb/C-50, 50 wt% of Nb_2_O_5_) prepared from niobium tartrate (Nb_2_O_5_) and glucose as raw materials via carbonization. The conversion of cellulose into HMF would increase by 77.8% from 53.3% in the THF/H_2_O system at 170°C for 8 h (Li et al., 2012).

### Selective Oxidation of HMF Into FDCA

HMF is regarded as a versatile platform compound and an intermediary that connects biomass resources and petrochemical industry (Zhang et al., 2015). It is a renewable furan chemical, which is produced from the dehydration of C_6_ carbohydrates. Except for its high value, HMF can be further transformed into diverse high-quality fuels such as C_9_-C_15_ alkanes and many oxidized furan chemicals (Wu et al., 2014; Zhang and Deng, 2015; Kang et al., 2019). For example, FDCA can take the place of the fossil resource (p-xylene), which was used for producing the polyesters. Currently, 98% of p-xylene produced was used as a raw material for the production of terephthalic acid, a monomer for polyethylene terephthalate (PET). PET is the fundamental material for the production of fibers, films, containers, packaging materials, molded articles, and household consumable goods (Zakrzewska et al., 2010; Fang et al., 2016).

### Noble Metal Catalysts

Metal is favored for the conversion of HMF to FDCA during the catalysis reaction. Noble metal catalysts offer an empty electron orbital, narrow level spacing, and diversity of coordination modes that can receive an electron pair from the reactants. The transfer of an electron pair results in bond weakening. These are the active sites for the reaction that is conducive to the adsorption of reactants on the catalysts. Gold-based catalysts were widely studied with different supports, and their reactivity was described as depending on the structure of the catalyst and the ratio of the gold nanoparticles with other metals or support (Jeong et al., 2015; Carrillo et al., 2018).

Oxidation of HMF in the presence of Ru catalysts and in the absence of base was found to be quantitative under moderate conditions. Different Ru catalysts were introduced with different supports, though some of them were not of much use because of metal leaching problems (Romero et al., 2016).

**Figure 6:**
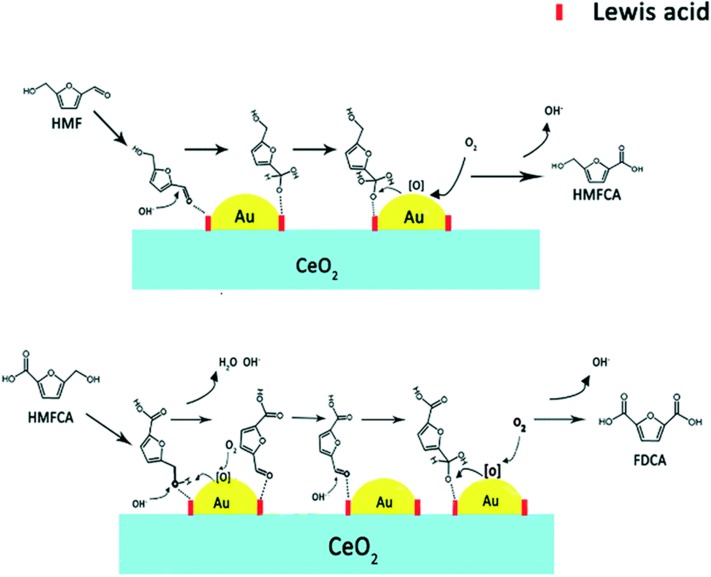
Possible reaction mechanism for oxidation of HMF to FDCA with the Au catalyst (take Au@CeO_2_ as an example). Used with permission of Royal Society of Chemistry, from Li Q. Q. et al. (2019) permission conveyed through Copyright Clearance Center, Inc.

The oxidation conversion of HMF into FDCA has mainly performed over noble metal (Pd, Pt, and Au) catalysts in an alkaline solution (Table 1). Single gold metal, though has an effective increase in the ratio of conversion, cannot totally convert HMF into FDCA. As Figure 6 shows, catalyst support was regarded as one oxygen pump by releasing and adsorbing the oxidizing species through the change of valence states during the redox process. And the molecular O_2_ was activated by the Au and promoted the formation of the carboxyl by releasing OH^−^ (Chen et al., 2013).

**Table 1:** Several catalysts for the oxidation of HMF into FDCA.

**No**.	**Catalyst**	Catalyst ratio^a*^	**Oxidant**	NaOH dosage^b*^	**T (K)**	**Time (h)**	**Con. (%)**	**Yield (%)**	**References**
1	Au/C	150	6.9 bar O_2_	2 equiv.	296	6	100	7	Davis et al., 2011
		150	20 bar O_2_	20 equiv.	296	22	100	72	Davis et al., 2011
2	Au/TiO_2_	150	20 bar Air	20 equiv.	295	22	100	79	Cai et al., 2013
3	Au-Pd/CNT	100	5 bar O_2_	No	373	12	100	94	Davis et al., 2012
4	Au/CeO_2_	400	50 bar Air	4 equiv.	443	4	100	90	Li Q. Q. et al., 2019
5	Au-Cu/TiO_2_	100	10 bar O_2_	4 equiv.	368	4	100	99	Wan et al., 2014
6	Au_8_-Pd_2_/C	200	10 bar O_2_	2 equiv.	333	6	>99	>99	Villa et al., 2013; Wan et al., 2014
7	Pt/C	100	6.9 bar O_2_	2 equiv.	296	6	100	79	Davis et al., 2011
8	Pt/C-EDA-4.1	100	10 bar O_2_	1.25 M	373	12	100	96	Han X. W. et al., 2016
9	Pt-Pd/C	100	10 bar O_2_	1.25 M	298	2	100	99	Davis et al., 2012
10	Pt/RGO	39	10 bar O_2_	5 equiv.	298	24	100	84	Negoi et al., 2014
11	Pd/C	150	6.9 bar O_2_	2 equiv.	296	6	100	71	Davis et al., 2011
12	Pd/TiO_2_	100	1 bar O_2_, 35 mL min^−1^	1.25 equiv.	363	8	>99	53	Davis et al., 2011
13	Pd/HT	21.2	1 bar O_2_, 35 mL min^−1^	1.25 equiv.	373	7	>99	99	Davis et al., 2011; Niu et al., 2014

a* *The catalyst ratio was defined as the molar ratio of HMF to the metals*.

b* *Equivalent calculated based on the molar ratio of NaOH to HMF*.

The Au/C catalysts can increase the yield of FDCA by 72% (Table 1, No. 1) (Davis et al., 2011). A different shape of the supporter has different catalysis efficiency. When TiO_2_ was used for supporting the Au as the catalyst, the yield of FDCA can reach up to 79% (Table 1, No. 2) (Cai et al., 2013). When gold metal added another kind of metal, the conversion ratio and yield reached above 94% (Davis et al., 2012). When support was changed by CeO_2_, the 90% yield was obtained after 4 h at 443 K under 20 bar O_2_, 4 equiv. (Li Q. Q. et al., 2019), NaOH condition (Table 1, No. 4). Adjustment of the molar ratio Au/Cu=1 in the Au-Cu/TiO_2_ catalyst is done by the immersion method. This kind of doping metal catalysis showed the highest catalytic activity, which has achieved a 99% FDCA yield at 368 K and 10 bar O_2_ (Table 1, No. 5) (Wan et al., 2014). This catalyst was stable without treating the leaching and aggregation for the metal nanoparticles. Two kinds of noble metal can be one step closer to increasing catalysis efficiency. Supported bimetallic Au–Pd catalysts appeared to have better catalytic performance than single Au catalysts (Villa et al., 2013). For example, an FDCA yield of 99% was produced after 4 h at 333 K over the Au_8_-Pd_2_/C catalyst (Table 1, No. 6). The Au_8_-Pd_2_/C catalyst could be reused for five runs with the FDCA yield remaining at 99% (Villa et al., 2013). In most cases, the oxidation of HMF into FDCA over Au catalysts was performed by the use of an excessive base.

The other important noble metal is Pt, and its catalysts are the subject of many theoretical, experimental, and industrial oxidation studies. Compared with Au, Pt has a higher yield of FDCA with the same C catalysis supporter under the same reaction condition. Verdeguer et al. (1994) studied Pt/C catalysts and the yield of FDCA was increased by 81% with full HMF conversion after 2 h at 25°C (Table 1, No. 7). The FDCA yield increased to 99% over a Pt-Pb/C catalyst. Kinetic studies showed that the first step was the oxidation of the formyl group, and then the hydroxymethyl group followed up. After 2 h at 25°C under 10 bar O_2_, the HMF was fully converted by Pt-Pd/C catalysts. However, the FDCA yield was 99% for Pt-Pd/C catalysts (Davis et al., 2012; Han X. W. et al., 2016). It means that the addition of Pd largely increased the catalysis reaction. Davis et al. (2011) reported the catalytic performance of Pt/C, Pd/C, and Au/C catalysts for HMF oxidation in 0.3 M NaOH solution at 23°C and 6.90 bar O_2_ pressure. The HMF conversion reached 100% after 6 h for these catalysts with FDCA and 5-hydroxymethyl-furan-2-carboxylic acid (HFCA) as the oxidation products. The yields of FDCA were 79% and 71% for Pt/C and Pd/C catalysts, respectively (Table 1, No. 7 and No. 11) (Han X. W. et al., 2016). However, because of the different reaction mechanisms, Au/C only has a 7% yield (Table 1, No. 1). Han X. W. et al. (2016) also investigated the Pt/C catalyst, with N doping in the Pt/C-EDA-4.1 catalyst showing the highest activity in the oxidation of HMF to FDCA, and as high as 96% FDCA was obtained under optimal reaction conditions (110 °C, 1.0 MPa O_2_, 12 h). With the addition of other noble metal, the yield of FDCA can be further increased by 99% for the Pt-Pd/C catalyst (Table 1, No. 8) (Davis et al., 2012). Besides the C catalyst supporter, reduced graphene oxide (RGO) was also used to stabilize Pt nanoparticles (Pt/RGO) for the oxidation of HMF at 25°C (Table 1, No. 10). FDCA was produced with a yield of 84%, while HFCA was produced with a yield of 16% by the Pt/RGO catalyst after 24 h under 10 bar O_2_ pressure (Negoi et al., 2014). The Pt/RGO catalyst efficiency was reused with 100% HMF conversion in each run. However, the FDCA yield showed a slight decrease with a slight increase in the HFCA yield. Several kinds of Pd catalysts were also active for the oxidation of HMF into FDCA. There was full HMF conversion in each run, but the FDCA yield decreased by 53% for Pd/TiO_2_ catalysis (Table 1, No.1 2) (Siyo et al., 2014). The Pd/HT catalyst also has a bond between Mg and metal with basic support, which was also active for the oxidation reaction of HMF into FDCA (Table 1, No. 13) (Liu et al., 2000). The yield of FDCA could reach up to 99% in water after 7 h at 100°C. And it has stable catalysis efficiency after the fourth run. There was only a slight decrease in the FDCA yield after the fifth run, while there was full HMF conversion over the Pd/HT catalyst.

#### Non-noble Metal Catalysts

**Figure 7:**
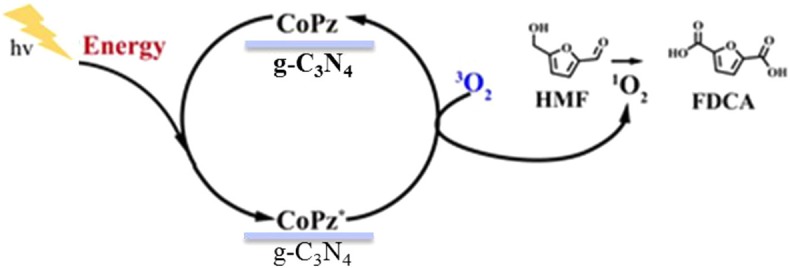
Possible mechanism of the photocatalytic oxidation of HMF into FDCA with the CoPz/g-C_3_N_4_ catalyst. Reprinted with permission from Xu et al. (2017) American Chemical Society.

Non-noble metallic oxides (such as Mn, Fe, Co, and Ni) (Jiang et al., 2016; You et al., 2016, 2017; Schade et al., 2018) have been developed to produce FDCA. A 90% FDCA yield, nearly 100% faradaic efficiency, and robust stability were achieved for NiCo_2_O_4_ nanowires (Gao et al., 2018). The non-noble metal (Fe–Zr–O) as a catalyst was used in [Bmim]Cl solvent for the conversion of fructose into HMF. And the formed HMF in the same reactor was further oxidized into FDCA. Under the one-pot conditions, an FDCA yield of 46.4% was obtained with fructose conversion of 100%. The potassium ferrate (K_2_FeO_4_) catalysis was employed, and a maximum of FDCA yield (48.3%) could be obtained under optimal reaction conditions (Zhang et al., 2015). Photocatalysis processes were usually used for improving the catalytic oxidation; CoPz/g-C_3_N_4_ is a highly efficient photocatalyst for the aerobic oxidation of HMF to FDCA under sunlight using air as an oxidant. As Figure 7 shows, the photocatalyst mechanism significantly enhanced the catalytic performance by activating O_2_ to ^1^O_2_ on the surface of CoPz and then selectively oxidized HMF into FDCA by the release of energy. Mn/N co-doped carbon supported Co catalysts from Co/Mn-lignin complexes, which exhibited excellent performance for aerobic oxidation of HMF to FDCA in water. After six times of use, the HMF conversion and FDCA yield are still 99.5 and 92.7% (Xu et al., 2017; Zhou et al., 2019).

Although many catalysts were developed, some internal disadvantages include high cost of noble metal catalysts, high energy consumption (because of the high reaction temperatures), and high oxygen pressures, and the requirement for the excessive base has been made up. Therefore, developing routes for the selective oxidation of HMF into FDCA under mild conditions is an important challenge. The design of effective, inexpensive, and nonprecious metal-based heterogeneous catalysts for selective HMF oxidation is still a challenge and is of enormous demand.

## Other Reactions

Although the most studied conversions of biomass platform chemicals are the focus on the above reactions, there are other several important reactions of biomass platform chemicals to fine chemicals. Depending on the different types of products, various processes, involving the precise cutting of different C–C bonds, are needed. Common types of reactions include isomerization, reforming, ketonization, or aldol condensation. In this section, recent advances of those reactions in the aqueous phase are discussed.

### Isomerization

**Figure 8:**
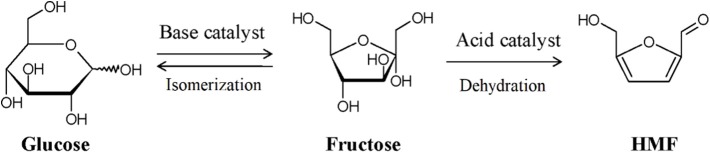
General process of LA production from cellulose. Used with permission of Royal Society of Chemistry, from Takagaki et al. (2009); permission conveyed through Copyright Clearance Center, Inc.

Because of the outstanding dehydration performance and subsequent utilization of ketose, the isomerization between aldose and ketose is an important research direction in the aqueous-phase conversion of biomass platform chemicals. In the production process of HMF or LA from cellulose, it has been proved that the dehydration of fructose (Figure 8) is much easier than the dehydration of glucose (Kang et al., 2018), and the isomerization is considered as the limiting step (Garces et al., 2019); therefore, improving the conversion and selectivity of the isomerization of glucose to fructose is a very important case for the utilization of these kinds of biobased chemicals.

The isomerization process of glucose to fructose can be accelerated by three types of catalysts, i.e., enzyme, base, and Lewis acid (Garces et al., 2019). Cordon et al. (2019) synthesized several hydrophobic Sn-Beta-F and hydrophilic Sn-Beta-OH catalysts and suggested that Sn-Beta-F can be activated first upon water exposure and then deactivated after 24 h. The characterization showed that the conversion of hydrophobic siloxane linkages into hydrophilic silanol defects within microporous voids led to the deactivation of Sn-Beta-F. They also suggested that the hydrogen-bonded networks formed by waters can confer the stability of the transition states and increase the conversion rates. Besides, they also suggested that the hydrolysis of the catalyst framework will lead to an increase in the hydrophilicity of its surface, which can decrease the isomerization conversion and the catalytic lifetime of the catalyst.

Relatively, Souzanchi et al. (2019) studied a series of solid base catalysts. The most active catalyst, MgO, showed 36.3% conversion of glucose and 22.8% yield of fructose at 100°C. Olson et al. (2019) using imogolite nanotubes, a naturally occurring aluminosilicate nanotube, as an isomerization catalyst achieved the highest conversion of 30% and selectivity of 45%. It can be seen that heterogeneous catalysts applied for the isomerization of glucose to fructose have obtained no perfect results and still need further research. To solve this, Garces et al. (2019) combined homogeneous and heterogeneous catalysts; the existence of Brønsted acids promoted the dehydration of fructose. That set up another approach since fructose is not the final product, and there is no need to be confined to the isomerization; combining several steps might have a better effect.

### Reforming

The aqueous-phase reforming (APR) reaction is an environmentally green process to convert polyols into H_2_ and alkanes or CO_2_ (Bastan et al., 2017). As a by-product of biodiesel production, the APR of glycerol is now an active area of research (Bastan et al., 2017); the main product is hydrogen. The APR process includes two main reactions: C–C cleavage (1) and water–gas shift (WGS) (2) (Guo et al., 2012).

(1) $$\begin{array}{r} {\text{C}}_{3}{\text{H}}_{8}{\text{O}}_{3}\to 3\text{CO}+4{\text{H}}_{2} \end{array}$$

(2) $$\begin{array}{r} \text{CO}+{\text{H}}_{2}\text{O}\to \text{C}{\text{O}}_{2}+{\text{H}}_{2} \end{array}$$

An ideal catalyst should be active both for these two reactions and should prevent the cleavage of C–O. VIII group metal catalysts, like Ni, Pd, and Pt, are commonly used. In recent studies, it has been shown that Pt-based catalysts have a high effect of hydrogen production (Callison et al., 2018), and γ-Al_2_O_3_ support showed a higher H_2_ producing rate than SiO_2_, TiO_2_, and CeO_2_ (Guo et al., 2012). Callison et al. (2018) used a Pt/Al_2_O_3_ catalyst to produce hydrogen or 1,2-propanediol. They discovered that the Pt particle size and the catalytic sites, such as the edge or the facet sites of Pt particles, can greatly affect the activity and selectivity of APR reaction. With Pt particle size in the range of 2–3.6 nm, even a slight change of Pt particle size can significantly affect the activity and selectivity of products. This phenomenon can be explained by surface chemistry: the edge sites like Pt (100), which are responsible for the dehydrogenation of glycerol, exist more on small Pt particles, and with the reaction proceeding, the edge sites decreased. In comparison, facet sites, such as Pt (111), are responsible for the dehydration of glycerol and tend to produce liquid products. This discovery suggests that the APR reaction or the competition between APR and dehydration not only depends on the species of the catalyst but also is very sensitive to the surface morphology and the particle size.

**Figure 9:**
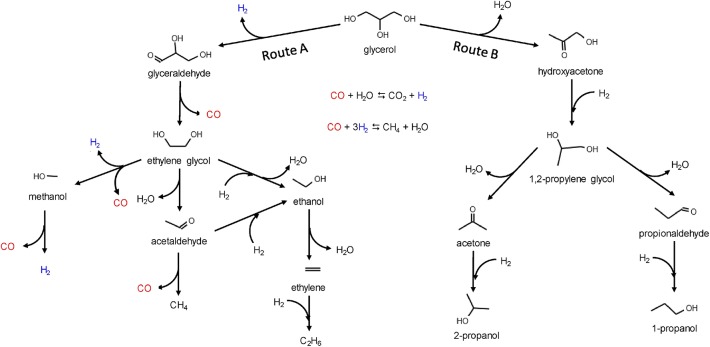
Reaction network for the glycerol APR on nickel catalysts. Reprinted from Morales-Marin et al. (2019), with permission from Elsevier.

Besides, Ni-based catalysts have attracted attention because of the low price and high activity in C–C scission (Morales-Marin et al., 2019). Morales-Marin et al. (2019) synthesized a nickel aluminate spinel by co-precipitation, and the reduction temperature of calcination was investigated, which indicated that the spinel structure is very stable. However, it was also found that hydrogen treatment could increase the density of medium-strength acid and basic sites, but could reduce the weak and strong sites that are both acid and basic. The reaction network comprises two routes as shown in Figure 9 (Morales-Marin et al., 2019), dehydrogenation (route A) and dehydration (route B), corresponding to the cleavage of C–C or C–O, respectively. Dehydrogenation requires metal sites, and dehydration requires acid sites. Analysis of the products indicated that the selectivity depended on the partial pressure of hydrogen, including the *in situ* formation of H_2_ by route A. The result showed that catalysts reduced at the highest temperatures produced the largest yield of H_2_, because of the preponderance of the activity of metal sites with respect to the activity of acid sites.

As shown in Figure 9, the APR process can also produce small chain alkanes, like methane or ethane. These products also have application foreground and research value. Bastan et al. (2017) used Ni/Ce_x_Zr_1−x_O_2_ to produce alkane. The XRD results showed that the support exists in a mixed-oxide form, and catalytic tests showed the catalytic performance of the catalyst related to the Ce/Zr atomic ratio. For the best performance achieved by 10% Ni/Ce_0.3_Zr_0.7_O_2_, it can produce 99% gas-phase production, with the selectivity of H_2_ and alkanes by 45 and 40%, respectively. This catalyst changes CO_2_ into alkanes while maintaining the H_2_ yield; further research might also focus on the conversion of other valuable chemicals.

### Ketonization

**Figure 10:**
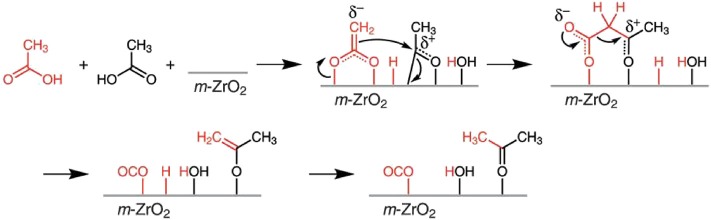
β-ketoacid route of acetic acid ketonization. Reprinted from Kumar et al. (2018), with permission from Elsevier.

Biobased chemicals are mostly composed of oxygenated compounds like alcohols, aldehydes, ketones, carboxylic acids, and aromatics. Take the hydrothermal liquefaction products of algae as an example; nearly 35% of total energy of the raw biomass and many other organic carbons have remained in the aqueous-phase product (Wu et al., 2017a). Acetic acid accounts for a large proportion, about 50% of the organic compounds in the aqueous phase. There is also a large amount of acetic acid that remained in the product of other biomass through thermochemical conversion (Wu et al., 2017a). Therefore, it is necessary to transform the biological acetic acid in the aqueous phase, which can significantly improve the utilization efficiency of algae, lignocellulosic, or other biomass resources. These molecules can be converted to ketones through ketonization and produce hydrocarbons that have a longer chain in the subsequent reaction. As reviewed by Kumar et al. (2018), the ketonization activity of carboxylic acid, aldehydes, alcohols, and esters was compared and sorted: carboxylic acid > aldehyde > alcohol > ester. The kinetically favored mechanism of carboxylic acid ketonization is the β-ketoacid route in Figure 10. The ketonization mechanism of other oxygenated compounds is not clear, but it was accepted that surface carboxylate intermediates are formed through an oxidation process under most conditions.

**Figure 11:**
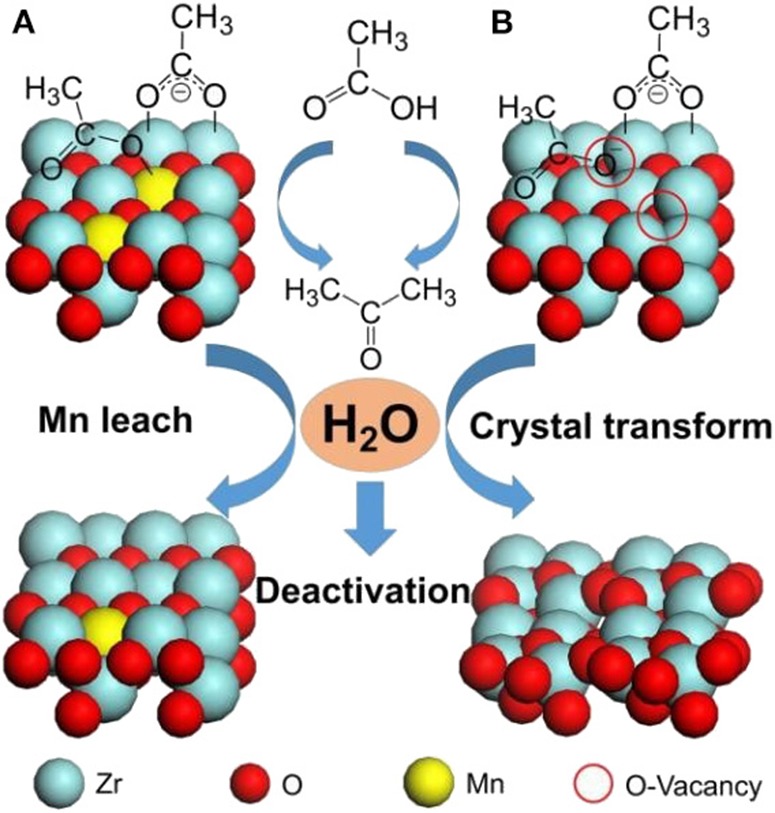
Aqueous-phase ketonization and deactivation process on tetragonal (011) facet of **(A)** ZrMn_y_O_x_ (y≥0.2) and **(B)** ZrMn_0_O_x_ catalysts. Reprinted from Wu et al. (2017a), with permission from Wiley.

Among ketonization of various oxygenated biomass chemicals, acetic acid is one of the most studied, and Zr-based catalysts are considered to be the most efficient. Wu et al. (2017a) synthesized a ZrMn_0.5_O_x_ catalyst, which showed the highest ketonization activity of 88.27% yield of acetone. It was found that Mn can easily form manganese carboxylate with the acetic acid, thus stabilizing the adsorption of acetic acid on the catalyst surface (Figure 11). The acid sites strongly affect the catalytic performance, so that the tetragonal ZrO_2_ phase, which was always accompanied by high acid property, was the main active phase. They also suggested two different mechanisms of deactivation, the leaching of Mn and the transformation from tetragonal ZrO_2_ to monoclinic ZrO_2_ (Figure 11).

Cai et al. (2018) studied the ketonization of acetic acid over a monoclinic ZrO_2_ catalyst by *ab initio* molecular dynamics (AIMD) simulations and density functional theory (DFT) calculations. Compared with vapor phase ketonization, water molecules showed both positive and negative effects on the reaction. On the one hand, water molecules hindered the reaction by blocking active sites or keeping acetic acid solvated through hydrogen bonds. On the other hand, it accelerated the proton transfer through the hydrogen-bonded water network and enhanced the protonation process. They identified an alternative mechanism that was more energetically favorable for the C–C bond formation; however, the α-hydrogen abstraction elementary step had high activation energy.

**Figure 12:**
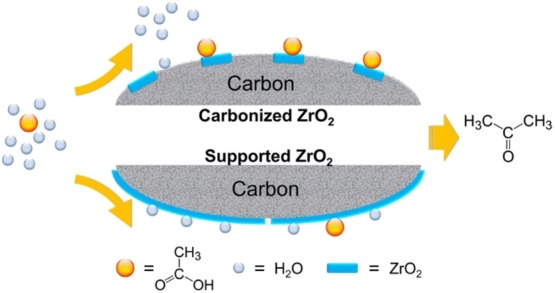
Comparison between the surface of high carbonized ZrO_2_ catalysts and conventional catalysts. Reprinted with permission from Wu et al. (2017b); American Chemical Society.

Because water has a blocking effect on the active site of the catalysts, the hydrophobic treatment of the catalyst surface can reduce the negative effect of water to a certain extent. Wu et al. (2017b) synthesized a series of highly carbonized ZrO_2_ catalysts. As shown in Figure 12, the carbon species in carbonized catalysts reduced the crystallite size of ZrO_2_ and improved the surface hydrophobicity, which can enrich the acetic acid on the catalyst surface and weaken the water adsorption, resulting in high ketonization activity. The leaching of carbon and the transformation of the crystal phase of ZrO_2_ are still the key factors of the catalyst deactivation, which is the direction of the improvement of heterogeneous catalysts for aqueous-phase ketonization.

In summary, there have been studies on the mechanism of oxygenated biomass chemical ketonization and some mechanisms have been widely accepted, but decisive evidence is still needed. Water phase transformation has been proved to have certain advantages; research on this has long-term significance for the utilization of biobased chemicals.

### Aldol Condensation

As mentioned above, ketonization and subsequent aldol condensation are a possible pathway to convert short-chain biomass platform chemicals into long-chain liquid hydrocarbon fuels or value-added chemicals. Although there are several ways to upgrade the primary biomass, the advantage of condensation is low carbon loss and by-product generation. As an important part of C–C bond formation and carbon chain growth, aldol condensation is usually catalyzed by alkaline catalysts (Chheda and Dumesic, 2007), ion-exchanged resins, or zeolites, and because of the high reactivity of aldehydes and ketones, the fractional aldol condensation is not easy to achieve, which makes the aldol condensation of stable platform chemicals very unique (Wu L. P. et al., 2016). Because of this, the aldol condensation of ketones with furfural or HMF has been extensively studied. Faba et al. (2012) studied the aldol condensation of HMF and acetone using MgAl and MgZr mixed oxide catalysts. The results showed that the ratio of medium-strength acid and basic sites can strongly affect the catalytic activity. The results suggested that C_9_ and C_15_ products are observed, and the selectivity of the C_15_ product decreased with the rise of conversion.

**Figure 13:**
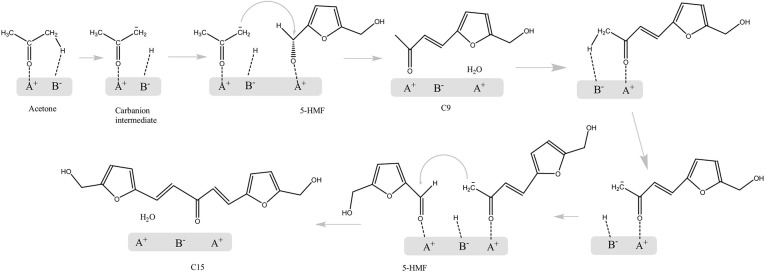
The aldol condensation mechanism of HMF and acetone. Reprinted from Cueto et al. (2017), with permission from Elsevier.

The aldol condensation mechanism of acetone and 5-HMF using MgAl and MgZr mixed oxide catalysts in the aqueous phase is studied by Cueto et al. (2017). As shown in Figure 13, a proton from acetone is first abstracted, and then attacks the carbonyl group of the HMF molecule (Cueto et al., 2017). The results also explained the selectivity of C_9_ and C_15_ varying with the conversion. A second aldolization of the C_9_ adduct can occur, as the C_9_ adduct still has an α-proton, but the large difference of activation energies between C_6_-, C_9_-, and C_15_-based reactions led to the selectivity difference with temperature and the total conversion.

Because C_5_ or C_6_ alkanes are difficult to be used as fuels due to the short carbon chain, the aqueous-phase aldol condensation is a promising route to convert C_5_ or C_6_ sugars that are directly produced from cellulose and hemicellulose as usable biofuels. However, there are not many studies about the application of these compounds in fuel manufacturing, which may be related to the relative value between fuels and chemicals. Though the direct pyrolysis or hydrothermal liquefaction of raw biomass as biofuels may be more competitive at present, it is undeniable that the aldol condensation of C_5_ or C_6_ platform chemicals has opened up a potential application route of upgrading the value of biomass and producing specific chemicals from biomass.

## Summary and Outlook

The aqueous-phase conversion of biomass platform chemicals over heterogeneous catalysts offers economic and environmental benefits to produce valuable chemicals from biomass. Besides, with water as a solvent, the principles of green chemistry are better embodied in terms of the reaction and separation processes. In this review, typical reactions such as dehydration, hydrogenation, oxidation, isomerization, reforming, ketonization, and aldol condensation have been introduced for a general conversion process of biomass. However, aqueous-phase catalytic conversion of biomass still faces many problems to be solved, and advances in the catalysts or the reactions themselves are still developing.

### Reaction Mechanisms and Kinetics

In the research of improving the conversion processes and catalysts, the reaction mechanisms are fundamentally needed. Compared with the complicated reaction system of the thermochemical conversion process of biomass to bio-oil, such as hydrothermal liquefaction or pyrolysis of the macromolecular biomass, the reaction mechanisms of conversions of biomass platform chemicals are quite simple. Despite this, catalysts with the same function may produce different products under different conditions. Taking the hydrogenation of furfural as an example, because of its high unsaturation, precise hydrogenation is not so easy to achieve, which makes it difficult to obtain a desirable product with high selectivity. Therefore, the precise regulation of products requires a better understanding of reaction mechanisms and kinetics. In recent years, in combination with the developing technical means, some breakthroughs have been made in the mechanism of many reactions. The common method is to combine kinetic studies with DFT calculation or isotopic labeling (Gilkey et al., 2015). The reaction order can be calculated by controlling the reaction time in a short time, which can reflect the participation level of various species in the reaction system and is helpful in inferring the reaction mechanism. DFT calculation can be used to calculate the energy of each transition state, judge the adsorption mode, and determine the pathway of the reaction process. Isotopic labeling can determine the transfer pathway of a certain kind of atom; e.g., using labeled hydrogen donor can track the hydrogen transfer during the hydrogenation step. In particular, the formation and fracture of chemical bonds and the sequence of competitive reactions can also be observed by labeling the atom in a specific site. However, the case in the aqueous phase is complicated, especially the influence of hydrogen bond and the fast proton exchange; studies are still needed at present, and further research on the mechanisms and kinetics of the reaction pathways will contribute to the development of this field.

### Catalysts Development

Hydrothermal stability is an eternal topic of heterogeneous catalysts in aqueous-phase conversion. The deactivation caused by structural damage or leaching of active components of catalyst pores is the main problem restricting the lifetime of catalysts. Previous efforts have been made in the past few years so that some catalysts with high hydrothermal stability have been formed. The treatment that has been reported to increase the hydrothermal stability of the heterogeneous catalysts includes doping heteroatoms, surface modification, and coating. In recent researches, most catalysts can be recycled, and no significant decrease in activity was observed in more than five times. This is a preliminary attempt at industrial application; the development of water-resistant catalysts with high activity and long-life cycle can be anticipated in the future. In addition, noble metal catalysts once had overwhelming efficiency advantages, but with the general increase in the yield led by the development of new catalysts, this advantage is no longer so obvious in some reactions. On the contrary, the expensive price is more prominent. Therefore, how to show its superiority in other aspects is important. The advantage may be reflected in a lower load and more mild reaction conditions. There is no doubt that all kinds of catalysts are still developing, and the criteria for evaluating the quality of catalysts are also diversified. It will be more and more difficult to make progress on the catalyst that is much better than the existing catalysts in all aspects. In the future, it might be a better way to select catalysts based on appropriate objectives.

### Economic Performance

Despite the fact that research on biomass aqueous-phase conversion to high value-added chemicals has shown environmental advantages, the economic feasibility is also one of the factors that ultimately affect the industrialization of it. In other words, if the existing technology cannot reflect economic advantages, its implementation will not be easy. Considering that higher heat capacity and more corrosiveness of water in contrast with traditional non-polar organic solvents like dioxane mean higher heating and equipment costs are needed, more researches of economic feasibility are still needed. In fact, that is not just a matter of cost and benefit; considering the transformation from the traditional petroleum-based industry, more social backgrounds need to be considered, which involves the formulation of relevant standards or the implementation of policies. It is not a chemistry or chemical engineering problem, but a socioeconomic problem. To solve this, research from a wider field is needed.

### Product Selection

Although the conversion of biomass as bio-oil is not covered in this article, bio-oil is also widely expected as a promising alternative energy. In fact, compared with the demand for high value-added chemicals, the demand for fuel is ever greater in human society. In other words, some biobased products will have a greater market and strategic significance as fuels. But on the other hand, because the petroleum industry has already had a very mature production process, the price of biobased fuels maybe not as high as that of chemicals at present. The combination of the production of high value-added chemicals with biobased fuels that have a large market and strategy can better reflect the value of biomass in both economic and strategic aspects. For these reasons, some more macroanalysis needs to be made in order to give a better guiding significance for the selection of utilizing route for biomass resources.

In this work, a series of aqueous-phase heterogeneous catalytic conversions of biomass platform molecules have been introduced, and the reaction mechanisms and signs of progress of catalysts have been highlighted. We hope readers can have a broad understanding of this promising field and be able to get guidance and technical support.

## Author Contributions

XL performed critical reviews and contributed to the section of Introduction, Hydrogenation, and Other Reactions. LZ and SW contributed to the preparation of the sections Oxidation and Dehydration, respectively. YW designed the structure of the manuscript and is responsible for the work. All authors discussed the results, wrote, and commented on the manuscript.

### Conflict of Interest

The authors declare that the research was conducted in the absence of any commercial or financial relationships that could be construed as a potential conflict of interest.
